# Barriers to HPV vaccine series completion among a predominantly hispanic border population: a mixed method evaluation

**DOI:** 10.1186/s13690-024-01344-y

**Published:** 2024-07-24

**Authors:** Amir Hernandez, Jessica Calderón-Mora, Hatty Lara, Nicole Drury, Jennifer Molokwu

**Affiliations:** 1 College of Health Science, University of Texas at El Paso, El Paso, Texas USA; 2 Department of Population Health, Dell Medical School, University of Texas at Austin, Austin, Texas USA; 3 Department of Psychology, University of Arizona College of Science, Tucson, Arizona USA; 4 Paul L. Foster School of Medicine, Texas Tech University Health Sciences Center El Paso, 5001 El Paso Dr., El Paso, Texas 79905 USA

**Keywords:** HPV, Mixed methods, Hispanic, Border population

## Abstract

**Background:**

Human Papillomavirus (HPV) infections are the most common sexually transmitted infections in the United States. The HPV vaccine is a vital tool to prevent against several cancers, namely cervical cancer. Unfortunately, the uptake of the HPV vaccine among Hispanics is relatively low. Some barriers to uptake include language barriers, cultural taboos, and cost.

**Purpose:**

This study aims to explore barriers to HPV vaccination in a predominantly Hispanic US-Mexico border county between June 2015 and March 2018.

**Methods:**

A mixed-method approach was used to analyze covariates associated with HPV vaccine uptake and to evaluate barriers to HPV vaccination from participant follow-up calls or reminder notes.

**Results:**

The total number of participants was 1,787. Young adults were less likely to complete the vaccination series than those aged 9-17, while individuals born in Mexico were more likely to do so. Failure to contact was the most common barrier (*n*=1,801, 86.42%), followed by scheduling concerns (*n*=99, 4.5%), being ineligible (74, 3.55%), completing series outside of the program (40, 1.92%), having medical concerns (36, 1.73%), and other reasons (34, 1.63%).

**Conclusion:**

We predominantly identified structural barriers and various health-related determinants regarding healthcare access and quality.

**Table:** 

Text box 1. Contributions to the literature
• There are limited studies evaluating the barriers to HPV vaccine uptake among a US-Mexico border population.
• Findings indicate that the most common barriers to HPV vaccine uptake in this population are much more structural and systemic.
• This information will better inform intervention development tailored to populations similar to ours who encounter systematic barriers such as language discordance, low socioeconomic status, and poor access.

## Background

The human papillomavirus (HPV) infection is the most common sexually transmitted infection in the United States (U.S.) and worldwide [1, 2]. It is known to cause most cervical cancer cases [3]. The HPV vaccine can prevent cancers of the cervix, vagina, vulva, penis, anus, rectum, and oropharynx [4]. After the introduction of HPV vaccines, cervical cancer incidence has decreased from 8.2 per 100,000 women in 2006 to 7.5 per 100,000 women in 2017 [5, 6]. Invasive cervical cancer cases significantly decreased among young women [7–12] between 2011 and 2017, indicating the success of the HPV vaccine in preventing cancer [6]. However, despite this national decrease in cervical cancer cases, women of Hispanic origin in the U.S. have a higher incidence and mortality rate than non-Hispanic white women (9.6/100,000 vs. 7.2/100,000 and 2.5/100,000 vs. 2.0/100,000, respectively) [13].

Despite the protection offered by the HPV vaccine against six types of cancer, data from the 2019 National Immunization Survey—Teen Survey indicates inadequate vaccine coverage [14]. The HPV vaccine has an initiation rate of 71.5%, with a completion rate of only 54.2% among adolescents [14]. This rate contrasts with other adolescent vaccines recommended at the same age, with vaccine coverage at 89.3% for at least one dose of the meningococcal vaccine and 90.1% for the Tetanus, Diphtheria, and Pertussis (Tdap) vaccine [15]. Among Hispanics, the rates of HPV vaccine uptake are about 87% among adolescents 13-17 years old, and only 35% of Hispanic women 18-26 years old have even started the vaccine series [16].

Some commonly reported barriers to HPV vaccination among Hispanics are a lack of knowledge and awareness of HPV and parental concerns that the HPV vaccine could increase sexual promiscuity in adolescents [7, 17–20]. Other barriers include safety concerns and a lack of provider recommendations. A meta-analysis found that Hispanic parents who have a higher perceived importance of the HPV vaccine as cancer prevention are more likely to have their children vaccinated [8]. Lack of health insurance and cost are also barriers among the Hispanic population. Finally, language barriers can impede appropriate dissemination of HPV education, and lack of language concordance between the provider and the patient can lead to mistrust of health information [7, 9, 17].

Few studies have evaluated the barriers to HPV vaccine uptake among a US-Mexico border population. Border populations are unique in that many individuals participate in cross-border healthcare to receive healthcare wherever it may be less expensive, leading to a lack of continuity in care [10]. As a result, it is challenging to study this type of population. Thus, our study fills a critical gap in the literature by providing information from a study directed at a border population. Our study aims to identify factors related to HPV vaccine uptake in a primarily Hispanic border population and describe themes of barriers among this group.

## Methods

### Study design and setting

This mixed-methods study used data from *Tiempo de Vacunarte* (Time to get Vaccinated). This multi-component intervention included outreach, education, navigation to services, and reduced access barriers with no-cost vaccines [11].

The program was delivered in El Paso, a county in West Texas on the US-Mexico border with an approximate population of 840,000, between June 2015 and March 2018. The population is mainly Hispanic, with socioeconomic challenges such as higher-than-average poverty rates and low health insurance coverage.

### Intervention

Those eligible for the study included individuals aged 18 to 26 years and parents or legal guardians of children ages 9 to 17 who had not initiated or completed the HPV vaccine series, were uninsured or underinsured, and had a Texas address. If individuals met all the criteria above but had insurance, they were enrolled in our navigation services only, in which staff followed up on their vaccination status with their regular doctor. Participants were recruited by *promotoras* (community health workers) from community centers, food banks, health fairs, community colleges, and trade schools. Our Institutional Review Board approved the study.

## Quantitative data

Once enrolled, the *promotora* administered a pre-survey and culturally tailored health education on HPV and the HPV vaccine in English or Spanish. The program’s educational component was developed and guided by the Health Belief Model (HBM) [12]. After completion of the education session, the post-survey was administered, and the eligible participant (child or young adult) received the no-cost HPV vaccine or was scheduled for administration later. In addition, we provided navigation services for all participants through reminder calls for follow-up doses. Each follow-up call was documented with conversation details, including barriers to scheduling and receipt of follow-up vaccinations.

### Measures

The pre-survey was comprised of demographic questions related to age, biological sex, ethnicity, educational attainment, married/living with a partner, annual household income, country of birth, and years in the U.S. Completion of the HPV vaccination series was defined as two doses for children aged 9 to 14 and three doses for those aged 15 to 26. The age at which the patient initiated and completed the series was considered when analyzing data.

### Data analysis

Demographic characteristics were displayed with descriptive statistics using mean and standard deviation (S.D.). We reported categorical data using frequencies and proportions. For the surveys, we used the child's information for age, gender, having a regular doctor, county of birth, ethnicity, length of time living in the U.S., and the parent's characteristics for other survey items. We used logistic regression models to estimate unadjusted odds ratios and 95% confidence intervals (CI) for factors associated with HPV vaccination series completion. Some of the factors assessed in the regression model include age, gender, ethnicity, education, married/living with a partner, annual household income, country of birth, and years in the U.S. Country of birth and years in the U.S. were separated by age group (children/adolescents and young adults). We conducted our statistical analyses using SAS 9.4.

## Qualitative data

The patient navigator assessed barriers to HPV vaccine appointment attendance and uptake during follow-up calls. These barriers were documented in the language of the call, and qualitative notes were translated into English by bilingual research staff.

### Method

The notes from the navigator contact section were exported from our program database into Microsoft Excel. The data was then extracted using the thematic analysis framework. Three research team members conducted a manual independent review of the qualitative data. Each team member analyzed the data and identified relevant themes. The themes emerging from the data were compiled based on individual analyses of team members. The emerging themes were then compiled into a codebook for all contact notes. The research staff and two research faculty members engaged in discussions about themes that were not consistent across the three separate lists. Inconsistencies were thoroughly discussed until a consensus was reached among the team members.

### Data analysis

After identifying the themes, the research team members reviewed the qualitative notes and organized them according to the appropriate themes using thematic analysis. After resolving any discrepancies, the team finalized the list of themes derived from the data, grouping the identified themes into five overarching categories or themes, providing a structured and comprehensive understanding of the data. The research team, consisting of the initial three members, collated the frequency of each theme. It's noted that a single note might contribute to the identification of more than one theme.

## Results

Between June 2015 and February 2018, our program recruited 2,380 eligible participants. Only one individual per family was included in the data analysis. The total sample size was 1,787 unique participants. The *Tiempo de Vacunarte* program had an overall initiation rate of 67.1% and a completion rate of 39.8% [11]. Table 1 shows the demographic characteristics of our participants. The mean age of the children/adolescents group (9-17 years old) was 12.2 years (SD=2.653). The majority of the children/adolescents were Hispanic (96.02%), female (53.75%), and born in the U.S. (73.19%). The mean age of the young adults (18-26 years) was 22.2 years (SD=2.745). The majority were also Hispanic (96.57%), female (72.45%), had a greater than high school education (85.53%), married (68.27%), and were born in the U.S. (64.84%). When comparing children/adolescents to young adults, the young adult age group had a higher proportion of females (72.45% vs. 53.75% *P*<.0001) and more non-Hispanic whites (3.11% vs. 1.64% *P*<.0001). Children were more likely to be born in the U.S. compared to adults (73.15% vs. 64.84%, *P*<.0001) and have resided in the US for less than or equal to 10 years (55.15% vs. 34.62% *P*<.0001).

**Table 1:** Demographic characteristics of Tiempo 1 participants, El Paso County, TX, 2015-2018 (*n*=1,787)

	**9-17 y.o. (*N*=854)**		**18-26 y.o. (*N*=933)**		***p*-value**
	**N**	**%**	**N**	**%**	
**Age,** *Mean, SD*	12.2 (2.653)		22.2 (2.745)		**<.0001**
**Biological Sex**					**<.0001**
Female	459	53.75	676	72.45	
Male	385	45.08	257	36.13	
*Missing*	10	1.17	0	0.00	
**Ethnicity**					**<.0001**
Hispanic/Latino	820	96.02	901	96.57	
Non-Hispanic White	14	1.64	29	3.11	
*Missing*	20	2.34	3	0.32	
**Education**					
Less than High School			133	14.26	
High School or More			798	85.53	
*Missing*			2	0.21	
**Married/Living with a Partner**					
No			637	68.27	
Yes			293	31.40	
*Missing*			3	0.33	
**Annual Household Income**					
Less than $20,000			279	29.90	
$20,000 or More			126	13.50	
Don't Know			501	53.70	
Refuse to Answer			25	2.69	
*Missing*			2	0.21	
**Country of Birth**					**<.0001**
United States	625	73.19	605	64.84	
Mexico	201	23.54	313	33.55	
Other	13	1.52	15	1.61	
*Missing*	15	1.75	0	0.00	
**Years in the U.S.**					**<.0001**
Less than or equal to 10 years	471	55.15	323	34.62	
11-19 years	381	44.61	197	21.11	
20 or more years	2	0.23	413	44.27	

Significance deemed as a *P*-value of ≤0.05

### Covariates of vaccine uptake

The unadjusted logistic regression exploring the covariates of HPV vaccine completion showed that young adults were less likely to complete the vaccination series (OR: 0.98, 95%CI: 0.96-0.99, *P*=0.012) compared to children/adolescents. However, in both age groups, we found that those born in Mexico were more likely to complete the vaccination series (young adults: OR: 1.40, 95% CI: 1.03-1.89, *P*=0.030 and children/adolescents: OR: 1.51, 95% CI: 1.03-1.89, *P*=0.013). Details of vaccine uptake covariates have been published previously (Molokwu et al., 2019). See Table 2.

**Table 2:** Unadjusted logistic regression analysis of HPV vaccination series completion covariates, El Paso County, TX, 2015-2018 (*N*=1,787)

**Characteristics**	**OR**	**95% CI**	***p*-value**
**Age**			
***9-17 years old [Children/Adolescents]***	*Reference*		
**18-26 years old [Young Adults]**	0.98	0.96-0.99	**0.012**
**Gender**			
***Female***	*Reference*		
**Male**	1.03	0.83-1.26	0.815
**Ethnicity**			
***Hispanic/Latino***	*Reference*		
**Non-Hispanic White**	0.82	0.61-1.10	0.182
**Education**			
***Less than High School***	*Reference*		
**High School or More**	0.98	0.94-1.10	0.163
**Married/Living with Partner**			
***No***	*Reference*		
**Yes**	0.91	0.66-1.25	0.568
**Annual Household Income**			
***Less than $20,000***	*Reference*		
**$20,000 or More**	0.93	0.69-1.25	0.641
**Don’t Know**	0.87	0.69-1.09	0.216
**Refuse to Answer**	0.52	0.21-1.30	0.162
**am**			
***United States***	*Reference*		
**Mexico**	1.51	1.09-2.09	**0.013**
**Other**	1.14	0.52-2.52	0.739
**Country of Birth – Young Adults**			
***United States***	*Reference*		
**Mexico**	1.40	1.03-1.89	**0.030**
**Other**	1.14	0.36-3.62	0.830
**Years in the U.S. – Children/Adolescents**			
***Less than or equal to 10***	*Reference*		
**11-19 years**	1.24	0.83-1.84	0.296
**20 or more years**	1	--	--
**Years in the U.S. – Young Adults**			
***Less than or equal to 10***	*Reference*		
**11-19 years**	1.11	0.83-1.49	0.467
**20 or more years**	1	--	--

*OR* Odds ratio, *CI* Confidence interval, Significance deemed as a *P*-value of ≤0.05

### Barrier themes

**Fig. 1:**
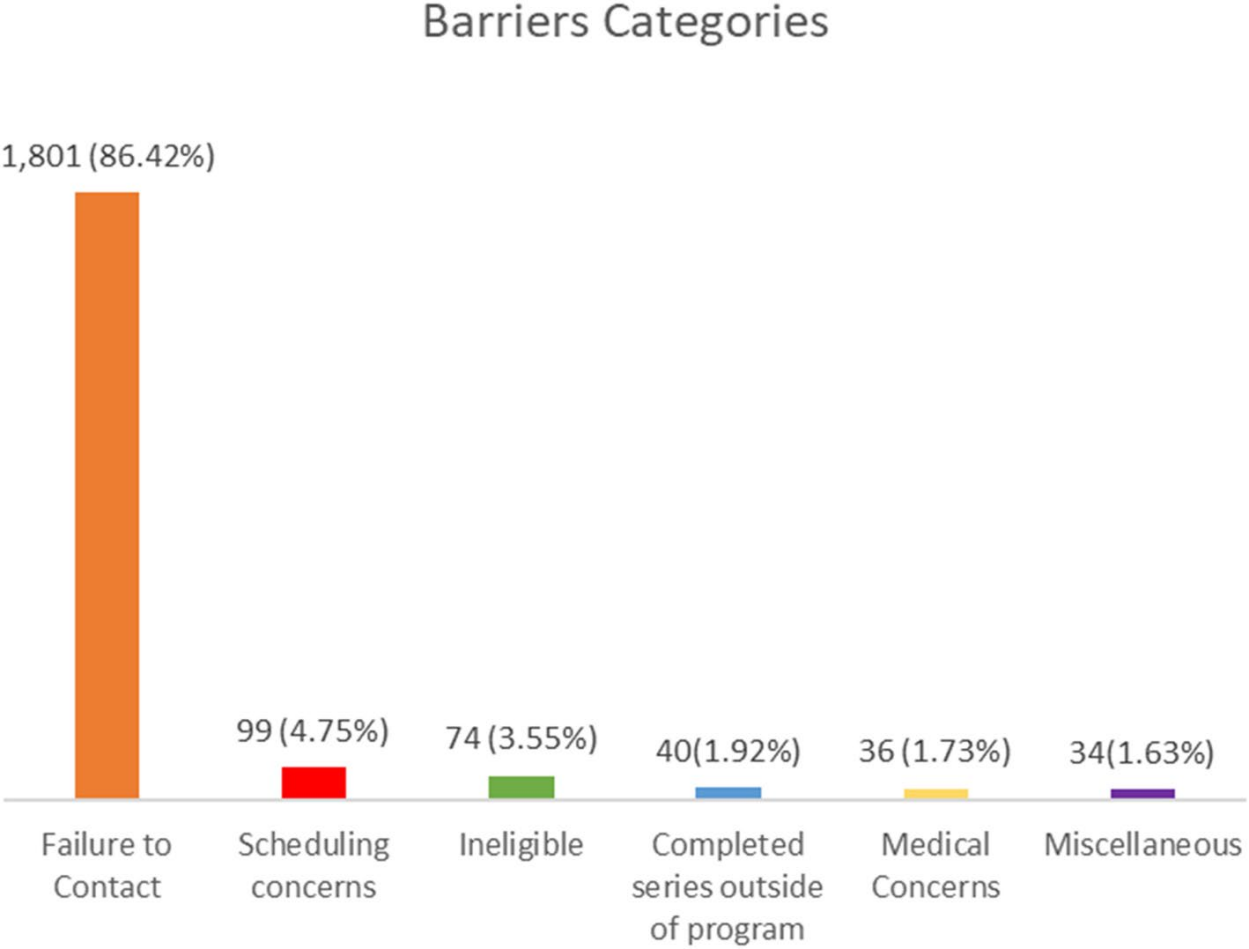
Frequency and percentage of barriers categories for Tiempo 1 participants, El Paso County, TX, 2015-2018 (*n*=2,084)

We examined the navigation notes from calls for *n*=1,047 participants with 2,084 navigation notes. The five barrier themes that emerged were failure to contact, scheduling concerns, ineligibility, completed series outside of the program, medical concerns, and miscellaneous (see Fig. 1). The average number of barriers identified per participant, regardless of age group, was 1.49.

#### Failure to contact

The inability to contact participants for follow-up was the most significant barrier our project staff faced in navigating participants to completion of the HPV vaccination series (*n*=1,801, 86.42%). As part of the program development, we identified the transient nature of our population as a possible barrier. We tried to ensure we had accurate contact information, such as collecting multiple contacts for each participant. Despite these efforts, we still had difficulty contacting many of our participants. The typical navigation comments documented included “*Phone number of participant and contact person both out of service” or when a response was received. “Person that answered phone stated that the phone number was not ‘participant name’. Participant’s contact provided the ‘correct’ phone number. However, when I called, there was no answer, and we were unable to leave a message”.* While also coded as failure to contact, there were several participants who chose to no longer engage with the program and hung the phone up when called “*Called to try to schedule the participant’s children’s last vaccine. Participant answered then hung up without talking to me” and* “*Patient hung up on me mid-sentence when I called again there was no answer.* We were, therefore, unable to identify any additional barriers in these participants.

#### Scheduling conflicts

Approximately 5% of contacts had difficulty scheduling follow-up appointments. The navigators identified scheduling difficulties, including conflicts with work schedules, school schedules, and being out of town on vacations, as well as other miscellaneous scheduling conflicts. Common scheduling comments identified in navigation notes include; “ *The Participant said that she has Tuesday and Wednesday off from work; however, it would be better if we scheduled in two weeks”;* “*Spoke with participant and she stated that she works late every day and that they will call us back to try to schedule an appointment for next week”;* “*The participant is interested in the vaccine, but she is now working out of town; she will call back when she is back”; “The child, ‘participant name,’ has many activities these days and is unavailable to keep an appointment. The mother then refused the vaccine for the child.”* These scheduling conflicts were sometimes overcome by scheduling home visits for vaccination, which is not always possible in real-world applications.

#### Ineligible participants

A total of 3.55% (*n*=74) were marked as ineligible at some point in the program. Again, it highlighted the difficulty in providing vaccines or other preventive services to a mobile and hard-to-reach population. Common reasons noted on navigation were no longer being uninsured or underinsured or moving out of town with such reports as “*The participant moved to New Mexico.”* Since funding for the program required participants to have a self-identified Texas address, they became ineligible if they moved to neighboring states.

#### Medical concerns

A small number of participants, about 2% (*n*=36), cited medical issues for not receiving the HPV vaccine. The most common medical concerns were participants who reported being pregnant following intake into the program. Navigation notes were documented; “*Participant name” is pregnant, will call back after she had the baby. “spoke with pt stated she is pregnant and cannot continue to vaccinate she will call us back after her baby is born to continue doses. “*

We did have a minimal number of participants who verbalized concerns about vaccine side effects they had heard from others or acute illness at the time of scheduling. While this was noted in navigation notes during scheduling, it was not identified as a barrier to receiving the vaccine.

#### Miscellaneous

The final 2% (*n*=34) of barriers were aggregated into a miscellaneous group that did not fit clearly into some of the other obstacles encountered by our navigators. These miscellaneous barriers were variable, such as “*I spoke with the mother, and she told me that her daughter is in jail and she doesn’t know when she will be out”* or another participant who reported, “Participant withdrew from the program and said she would no longer sign anything. She indicated that someone stole her identity and had privacy concerns”.

#### Completed vaccine series externally

This was not considered a proper barrier as we had participants in the program report completion or intention to continue the vaccine series with *other providers**** (***Approximately 2%). “*The mother said she will bring in ‘participant name’ to receive the third dose today. However, the mother later called back to say that she took the child to the pediatrician, and they administered the vaccine there.”* However, some indicated that they were discouraged by their provider and told only to get vaccines from a doctor’s office. *“Participant was called and denied our program due to her doctor advising her to only get vaccines at the doctor’s office.”*

## Discussion

In our multi-component, culturally appropriate HPV vaccination program to promote uptake, we found that children/adolescents and those born in Mexico were more likely to be vaccinated. However, it was also determined that the most common barrier to the HPV vaccine in our U.S.-Mexico border population was loss-to-follow up due to changing contact information or simply not answering calls from program staff.

The common barriers to HPV vaccine uptake in *Tiempo de Vacunarte* were much more structural than those noted in previous studies, primarily due to a lack of knowledge and misinformation [21]. Therefore, for this population along the US-Mexico border, social determinants of health (SDOH) related to healthcare access, quality, and economic stability must be addressed to improve vaccine uptake [22]. Our program attempted to overcome structural barriers by having a certified medical assistant on staff who could administer the vaccines on-site when an individual was recruited. We also offered home visits as a last resort; however, this did not necessarily translate to an increase in the completion of the HPV vaccine series. These identified barriers would need to be addressed at the policy level. In addition, structural barriers, such as lack of transportation, affordable means of communication, work hour flexibility, and childcare affecting individuals' ability to schedule health care visits, would need to be considered when developing similar programs or interventions. Therefore, ways of limiting or reducing structural barriers to vaccination were heavily considered when designing program methods for the next grant cycle.

Although HPV vaccination initiation rates are higher among Hispanic populations, the completion of the series is not quite as high. This rate can be improved through community programs that provide direct outreach to the community, especially those who do not have a regular doctor. Through lessons learned from this program, we have incorporated more of a social media presence to provide additional communication and health education to participants and the community. We have also developed a voucher system with a national pharmacy that allows our participants to obtain their vaccines at any location most convenient to them with no appointment.

Aside from what was mentioned above, other strengths of our study include providing all health education in both English and Spanish and ensuring that the project staff are bilingual. In addition, our *promotoras* were from a community similar to the priority population and, therefore, were relatable to the participants, which aided in working through barriers related to concerns. The major strength of this study is that it is the first study, to our knowledge, to highlight barriers directly related to social determinants of health that impede completion of the HPV vaccine series among a border population.

A limitation of our study is the transient nature of a significant proportion of our population. We attempted to counter this by requesting multiple contact numbers, including contact information for a family member or friend, but these individuals often would not respond. Another limitation is that we did not have any partnerships with local community clinics or pharmacies to administer the follow-up vaccines in a more convenient avenue for participants. Finally, our program's eligibility was focused on uninsured and underinsured individuals, so this may not represent the entire Hispanic border population.

Despite the barriers to uptake of the HPV vaccine among our Hispanic border population identified through our study, it is essential to note that this evidence-based approach resulted in the provision of 3,192 HPV vaccine doses with an overall initiation rate of 67.1% and a completion rate of 39.8% [11].

## Conclusion

Our study emphasizes the importance of integrating components in vaccination programs that directly address the social determinants of health-related to healthcare access, quality, and economic stability. This information will better inform intervention development tailored to populations similar to ours who encounter systematic barriers such as language discordance, low socioeconomic status, and poor access to increase the HPV vaccine series completion rates. Findings from our study also suggest that clinics need to ensure their policies include the HPV vaccine being discussed with all age-eligible patients and that there is a notification in the electronic health records to remind providers to provide the vaccine recommendation.

## Data Availability

No datasets were generated or analysed during the current study.
